# Indoor environment assessment of special wards of educational hospitals for the detection of fungal contamination sources: A multi-center study (2019-2021)

**DOI:** 10.32598/CMM.2023.1370

**Published:** 2022-12

**Authors:** Mona Ghazanfari, Jamshid Yazdani Charati, Nasser Keikha, Mahdi Kholoujini, Firoozeh Kermani, Yaser Nasirzadeh, Behrad Roohi, Mohammad Hassan Minooeianhaghighi, Bahram Salari, Seyed Ali Jeddi, Mojtaba Didehdar, Azar Shokri, Sekhavat Ameri Seyahooei, Narges Aslani, Mehdi Nazeri, Aynaz Ghojoghi, Kazem Amirizad, Maryam Azish, Mohsen Nosratabadi, Mohammad Reza Zakerian, Shakiba Hedayati, Hedieh Hatamipour, Mahdi Abastabar, Iman Haghani, Mohammad T. Hedayati

**Affiliations:** 1 Invasive Fungi Research Center, Communicable Diseases Institute, Mazandaran University of Medical Sciences, Sari, Iran; 2 Department of Medical Mycology, Faculty of Medicine, Mazandaran University of Medical Sciences, Sari, Iran; 3 Department of Biostatistics, Faculty of Health, Mazandaran University of Medical Sciences, Sari, Iran; 4 Infectious Disease and Tropical Medicine Research Center, Research Institute of Cellular and Molecular Sciences in Infectious Diseases, Zahedan University of Medical Sciences, Zahedan, Iran; 5 Beheshti Hospital, Hamadan University of Medical Sciences, Hamadan, Iran; 6 Department of Medical Microbiology, Faculty of Medicine, Infectious Diseases Research Center, Gonabad University of Medical Sciences, Gonabad, Iran; 7 Department of Laboratory Sciences, School of Allied Sciences, Abadan University of Medical Sciences, Abadan, Iran; 8 Department of Medical Parasitology and Mycology, School of Medicine, Arak University of Medical Sciences, Arak, Iran; 9 Vector-Borne Diseases Research Center, North Khorasan University of Medical Sciences, Bojnurd, Iran; 10 0Infectious and Tropical Diseases Research Center, Hormozgan Health Institute, Hormozgan University of Medical Sciences, Bandar Abbas, Iran; 11 1Infectious and Tropical Diseases Research Centre, Tabriz University of Medical Sciences, Tabriz, Iran; 12 2Department of Medical Parasitology and Mycology, Faculty of Medicine, Kashan University of Medical Sciences, Kashan, Iran; 13 3Department of Medical Mycology, School of Medicine, Ahvaz Jundishapur University of Medical Sciences, Ahvaz, Iran; 14 4Department of Mycology, Faculty of Medical Sciences, Tarbiat Modares University, Tehran, Iran; 15 5Department of Medical Parasitology and Mycology, School of Medicine, Dezful University of Medical Sciences, Dezful, Iran; 16 6Department of Medical Laboratory Sciences, Sirjan Faculty of Medical Sciences, Sirjan, Iran; 17 7Shohada Hospital-Gonbad, Golestan University of Medical Sciences and Health Services, Gorgan, Iran; 18 8Student Research Committee Center, Mazandaran University of Medical Sciences, Sari, Iran

**Keywords:** Airborne fungi, Hospital, Indoor air, Equipment, Sources of fungal contamination

## Abstract

**Background and Purpose:**

The hospital environment was reported as a real habitat for different microorganisms, especially mold fungi. On the other hand, these opportunistic fungi were considered hospital-acquired mold infections in patients with weak immune status. Therefore, this multi-center study aimed to evaluate 23 hospitals in 18 provinces of Iran for fungal contamination sources.

**Materials and Methods:**

In total, 43 opened Petri plates and 213 surface samples were collected throughout different wards of 23 hospitals. All collected samples were inoculated into Sabouraud Dextrose Agar containing Chloramphenicol (SC), and the plates were then incubated at 27-30ºC for 7-14 days.

**Results:**

A total of 210 fungal colonies from equipment (162, 77.1%) and air (48, 22.9%) were identified. The most predominant isolated genus was *Aspergillus* (47.5%), followed by *Rhizopus* (14.2%), *Mucor* (11.7%), and *Cladosporium* (9.2%). *Aspergillus* (39.5%), *Cladosporium* (16.6%), as well as *Penicillium* and Sterile hyphae (10.4% each), were the most isolates from the air samples. Moreover, intensive care units (38.5%) and operating rooms (21.9%) had the highest number of isolated fungal colonies. Out of 256 collected samples from equipment and air, 163 (63.7%) were positive for fungal growth. The rate of fungal contamination in instrument and air samples was 128/213 (60.1%) and 35/43 (81.2%), respectively. Among the isolated species of *Aspergillus*, *A. flavus* complex (38/96, 39.6%), *A. niger* complex (31/96, 32.3%), and *A. fumigatus* complex (15/96, 15.6%) were the commonest species.

**Conclusion:**

According to our findings, in addition to air, equipment and instrument should be considered among the significant sources of fungal contamination in the indoor environment of hospitals.

## Introduction

Hospital-acquired mold infections (HAMI) are among significant healthcare problems engendering great challenges to the healthcare systems. Among the main reasons for the rise of HAMI, increasing the number of the susceptible host because of an increase in patients with solid organ transplants, hematopoietic stem cell transplants, long stays in intensive care units (ICU), intubation, and utilizing broad-spectrum antibiotics have been more considered [ 1 , 2 ]. *Aspergillus* and some species in the order of Mucorales were reported as the main causative agent of HAMI [ 3 , 4 ]. After the COVID-19 pandemic, mold infections, especially invasive aspergillosis and mucormycosis, were reported to be among the most common complications with high mortality in COVID-19 patients admitted to the ICU [ 5 , 6 ]. Following that, the monitoring of the indoor environment of the hospital in terms of fungi was seriously emphasized [ 7 - 9 ].

Indoor air, equipment, medical devices in operating rooms, ICU, and specific units with patients with high risk for invasive fungal infections including hematological malignancies or immunosuppress conditions are attracted to transfer the fungi to susceptible patients. It has been stated that likely sources of mold fungi, especially *Aspergillus* and *Fusarium* species in the indoor environment of the hospital are conditioning systems, decomposed organic matter, dust, ornamental plants, food, water, and, in particular, construction in hospitals and their surrounding areas or the entry of outdoor fungal spores from the outside into the hospital. [ 10 , 11 , 12 ]. The negligence of hygienic and protective issues in the hospital leads to the occurrence of outbreaks of life-threatening mold infections [ 1 ]. 

It has been suggested that healthcare equipment and the indoor environment are among the most common reservoirs of etiological agents associated with hospital-acquired infections [ 13 ]. On the other hand, it is important to note that the reservoirs of the etiological agent are among the most important links in the chain of the infectious disease process [ 13 ]. Hence, recognizing the distribution and determination of the contamination sources of different fungi in the indoor environment of a hospital may be helpful in a preventive strategy of HAMI. Therefore, this multi-center study aimed to evaluate 23 hospitals in 18 provinces of Iran for fungal contamination sources.

## Materials and Methods

### Sampling sites

The present descriptive study was carried out in different educational hospitals from different provinces of Iran from September 2020 to January 2022. The samples were taken from the surfaces of equipment and appliances (e.g., computers, ventilators, telemeters, pacemakers, anesthesia machines, and endotracheal tubes, as well as sinks, floors, and walls) in different wards including ICU, neonatal ICU (NICU), operating room, neonatal, general, and oncology. In each ward, indoor air was also evaluated for airborne fungal spores. All samples were collected from all hospitals from 8 to 12 AM.

### Surface sampling

The tubes containing a cotton swab and PBS+0.1% tween 20 (2-3 ml) (PBS: KCL: 0.2 g, KH2PO4: 0.2 g, NaCl: 8 g, Na2HPO4: 1.15 g with pH: 7.4) were prepared and sterilized. Surface samples were collected by pressing the sterile moisturized cotton swab on each of the different areas of equipment and appliances, or the surface of walls and returned into the tube. Subsequently, the tubes were quickly transferred to the laboratory. Each tube contained a swab that was separately shaken by a shaker (Techne, UK) for 1 min, and after that, the swab was safely discarded, and the tube was centrifuged for 10 min at 3000 rpm. The supernatant was discarded, and the sediment was vortexed for 30 sec, and then, 20µl of each sample was inoculated into Sabouraud Dextrose Agar (QUELAB, Montreal, Quebec, Canada) containing Chloramphenicol (SC), separately. The plates were incubated at 27-30ºC for 7-14 days. Air sampling

The airborne fungal spores were collected by the sedimentation method. Opened Petri dish plates containing SC were used at the level of breathing height from 1 to 1.5 m for each room for 15 min. The collected plates were incubated at 27-30ºC for 7-14 days.

### Species identification

The grown fungi were identified by standard mycological techniques based on gross cultural and microscopic morphology. The fungi that could not be identified in this manner were sub-cultured on potato dextrose agar (Condalab, Madrid, Spain), water agar HiMedia, India), and /or slide cultures for further study. The *Aspergillus* species were identified by subculture onto the Czapek Dox Agar medium (Condalab, Madrid, Spain) and described according to the macroscopic and microscopic characteristics of each colony. These were then identified at the species level using keys by Raper and Fennel [ 14 ]. Based on these keys, species identification not only depended on colony characteristics and the morphology of conidiophore including conidial head, vesicle, conidiogenous cell, and conidia but also on the characteristics of the cleistothecia, sclerotia, and Hülle cells if they were present.

### Data Analysis

The airborne fungal spore levels were analyzed from descriptive statistics (SPSS 25, IBM, USA). The fungal contamination rate of each hospital located in different cities was calculated using the below formula:

$\mathrm{Fungal contamination rate}=\frac{\mathrm{Number of positive collected samples}}{\mathrm{Total number of collected samples}}\times 100$

## Results

During the study, 23 hospitals from 18 cities in different parts of Iran were checked for indoor fungal contamination. These cities were the capital of different provinces of Iran, except for Abadan and Dezful which are the cities near Ahvaz, the capital of Khuzestan province. In total, 213 and 43 plates containing SC medium were prepared from equipment and air, respectively. In each city, the main educational hospital was evaluated, except for two provinces of Mazandaran and Zahedan, where 4 and 3 hospitals were evaluated, respectively. It should be mentioned that air samples could not be obtained from Ilam, Zahedan, Tabriz, Birjand, Abadan, Dezful, and Mashhad. A total of 256 samples were collected from equipment and air. Moreover, 210 fungal colonies from equipment (162, 77.1%) and air (48, 22.9%) were identified. Generally, *Aspergillus* (96/210, 45.7%) was the most prevalent isolated genus. In equipment samples, the genus *Aspergillus* was also the most common isolate (77/162, 47.5%), followed by the genera *Rhizopus* (23/162, 14.2%), *Mucor* (19/162, 11.7%), and *Cladosporium* (15/162, 9.2%). 

*Aspergillus* (19/48, 39.5%), *Cladosporium* (8/48, 16.6%), as well as *Penicillium* and Sterile hyphae (5/48, 10.4% each), were the most isolates from the air collected samples (Table 1). 

**Table 1:** Distribution of the isolated fungi from different wards of the studied hospitals

	Devices						Total n (%)	Air						Total n (%)	Total n (%)
	ICU	NICU	Operating room	Neonatal	Oncology	General		ICU	NICU	Operating room	Neonatal	Oncology	General		
*Aspergillus*	24	1	21	6	13	12	77(47.5)	6	1	1		6	5	19(39.5)	96(45.7)
*Rhizopus*	8	2	9		3	1	23(14.2)	1						1(2.1)	24(11.4)
*Mucor*	8	1	3	1	4	2	19(11.7)	1		1				2(4.2)	21(10)
*Cladosporium*	7	1	2		1	4	15(9.2)	4	1	1		2		8(16.6)	23(10.9)
*Penicillium*	4		3		1		8(4.9)	2	2			1		5(10.4)	13(6.2 )
*Alternaria*	3		1				4(2.5)	1						1(2.1)	5(2.4)
*Trichoderma*	2					2	4(2.5)			2				2(4.2)	6(2.9)
Sterile hyphae				2		1	3(1.9)	1			3		1	5(10.4)	8(3.8)
*Fusarium*	1		2				3(1.9)							0	3 (1.4)
*Neoscytalidium*	2						2(1.2)	1						1(2.1)	3(1.4)
*Trichosporo*n							0	2						2(4.2)	2(0.9)
*Paecilomyces*					1		1(0.6)							0	1(0.5)
*Drechslera*	1						1(0.6)							0	1(0.5)
*Syncephalastrum*	1						1(0.6)							0	1(0.5)
*Trichothecium*	1						1(0.6)							0	1(0.5)
*Exophilia*							0		1					1(2.1)	1(0.5)
*Nigrospora*							0		1					1(2.1)	1(0.5)
Total	62(29.5)	5(2.4)	41(19.5)	9(4.3)	23(10.9)	22(10.5)	162(100)	19(9.0)	6(2.9)	5(2.4)	3(1.4)	9(4.3)	6(2.9)	48(100)	210(100)

Totally, out of the 162 and 48 fungal colonies isolated from equipment and air samples, 47.5% and 39.5% were related to *Aspergillus*, respectively. Table 2 shows in detail the distribution of different fungi isolated from different hospitals located in different cities according to the collected samples from equipment and air. In general, *Aspergillus* species were the main isolated mold fungi in Arak (8/8, 100%), Ilam (10/12, 83.3%), Zahedan (14/18, 77.7%), and Ardabil (8/11, 72.7%). The lowest prevalence of *Aspergillus* was observed in Tabriz (0/6), Tehran (1/7, 14.2%), and Abadan (1/6, 16.7%). *Cladosporium* species were found only in Sari (13/54, 24%), Bojnurd (4/14, 28.5%), Hamadan (2/8, 25%), Isfahan (3/21, 14.2%), and Gorgan (1/10, 10%). *Rhizopus* species was the most predominant genus in Tabriz (4/6, 66.6%). *Penicillium* species had a higher rate in Tabriz (2/6, 33.3%) and Tehran (2/7, 28.5%). The highest rates of isolation of *Mucor* species were observed in Tehran (4/7, 57.1%) and Abadan (5/6, 83.3%), compared to other cities. 

**Table 2:** Distribution of the isolated fungi from different hospitals located in different provinces of Iran

City (number of collected samples)		Devices						Total n (%)	Air						Total n (%)	Total n (%)
	Isolated fungi	ICU	NICU	NICU Operating room	Neonatal	Neonatal Oncology	General		ICU	NICU	Operating room	Neonatal	Oncology	General		
Sari (Device: n=36; air: n=12)	*Aspergillus spp.*	3		2	1	2	2	10(23.8)					1		1(8.3)	11(20.4)
	*Alternaria*	1		1				2(4.8)							0	2(3.7)
	*Fusarium*			1				1(2.4)							0	1(1.8)
	*Rhizopus*			6		1	1	8(19.0)							0	8(14.8)
	*Cladosporium*	5	1	2		1		9(21.4)	2		1		1		4(33.3)	13(24.0)
	*Penicillium*	3				1		4(9.5)							0	4(7.4)
	*Trichosporon*							0	2						2(16.7)	2(3.7)
	*Mucor*					1		1(2.4)							0	1(1.8)
	*Paecilomyces*					1		1(2.4)							0	1(1.8)
	*Neoscytalidium*	2						2(4.8)							0	2(3.7)
	*Sterile hyphae*				2		1	3(7.1)	1			3		1	5(41.7)	8(14.8)
	*Trichoderma*						1	1(2.4)							0	1(1.8)
	Total	14	1	12	3	7	5	42(100)	5	0	1	3	2	1	12(100)	54
Ilam (Device: n=9; air: n=0)	*Aspergillus spp.*	1		5	2		2	10(83.3)							0	10(83.3)
	*Fusarium*			1				1(8.33)							0	1(8.33)
	*Drechslera*	1						1(8.33)							0	1(8.33)
	Total	2	0	6	2	0	2	12(100)							0	12
Zahedan (Device: n=27; air: n=0)	*Aspergillus spp.*	2		6		4	2	14(77.8)							0	14(77.7)
	*Mucor*					1	1	2(11.1)							0	2(11.1)
	*Rhizopus*	1		1				2(11.1)							0	2(11.1)
	Total	3	0	7	0	5	3	18(100)							0	18
Bojnurd (Device: n=9; air: n=3)	*Aspergillus spp.*	4		1		2		7(63.6)	1				1		2(66.7)	9(64.2)
	*Rhizopus*					1		1(9.1)							0	1(7.1)
	*Cladosporium*	1					2	3(27.3)	1						1(33.3)	4(28.5)
	Total	5	0	1	0	3	2	11(100)	2	0	0	0	1	0	3(100)	14
Hamadan (Device: n=9; air: n=3)	*Aspergillus spp.*	2				1		3(75)							0	3(37.5)
	*Rhizopus*					1		1(25)							0	1(12.5)
	*Mucor*							0	1		1				2(50)	2(25)
	*Cladosporium*							0	1				1		2(50)	2(25)
	Total	2	0	0	0	2	0	4(100)	2	0	1	0	1	0	4(100)	8
Tehran (Device: n=9; air: n=3)	*Mucor*	3				1		4(80)							0	4(57.1)
	*Aspergillus spp.*	1						1(20)							0	1(14.3)
	*Penicillium*							0	1				1		2(100)	2(28.5)
	total	4	0	0	0	1	0	5(100)	1	0	0	0	1	0	2(100)	7
Dezful (Device: n=9)	*Aspergillus spp.*	1						1(25)							0	1(25)
	*Trichoderma*	2						2(50)							0	2(50)
	*Alternaria*	1						1(25)							0	1(25)
	Total	4	0	0	0	0	0	4(100)								4
Abadan (Device: n=9)	*Mucor*	1		3		1		5(83.3)							0	5(83.3)
	*Aspergillus spp.*	1						1(16.7)							0	1(16.7)
	Total	2	0	3	0	1	0	6(100)							0	6
Isfahan (Device: n=15; air: n=4)	*Mucor*	2	1		1			4(30.8)							0	4(19)
	*Rhizopus*	1	1					2(15.4)							0	2(9.5)
	*Aspergillus spp.*	2	1		1			4(30.8)		1					1(12.5)	5(23.8)
	*Syncephalastrum*	1						1(7.6)							0	1(4.7)
	*Penicillium*							0	1	2					3(37.5)	3(14.2)
	*Cladosporium*	1					1	2(15.4)		1					1(12.5)	3(14.2)
	*Alternaria*							0	1						1(12.5)	1(4.7)
	*Exophilia*							0		1					1(12.5)	1(4.7)
	*Nigrospora*							0		1					1(12.5)	1(4.7)
	Total	7	3	0	2	0	1	13(100)	2	6	0	0	0	0	8(100)	21
Ardabil (Device: n=9; air: n=3)	Aspergillus spp.	3		2			2	7(87.5)						1	1(33.3)	8(72.7)
	Mucor						1	1(12.5)							0	1(9.1)
	Trichoderma							0			1				1(33.3)	1(9.1)
	Neoscytalidium							0	1						1(33.3)	1(9.1)
	Total	3	0	2	0	0	3	8(100)	1	0	1	0	0	1	3(100)	11
Tabriz (Device: n=9; air: n=0)	*Penicillium*	1		1				2(33.3)							0	2(33.3)
	*Rhizopus*	2		2				4(66.6)							0	4(66.6)
	Total	3	0	3	0	0	0	6(100)							0	6
Rasht (Device: n=9; air: n=3)	*Mucor*	2						2(25)							0	2(20)
	*Aspergillus spp.*	2		1			1	4(50)						2	2(100)	6(60)
	*Fusarium*	1						1(12.5)							0	1(10)
	*Trichoderma*						1	1(12.5)							0	1(10)
	Total	5	0	1	0	0	2	8(100)	0	0	0	0	0	2	2(100)	10
Gorgan (Device: n=9; air: n=3)	*Aspergillus spp.*	1				1	1	3(60)	1		1			2	4(80)	7(70)
	*Trichoderma*							0			1				1(20)	1(10)
	*Penicillium*			1				1(20)							0	1(10)
	*Cladosporium*						1	1(20)							0	1(10)
	Total	1	0	1	0	1	2	5(100)	1	0	2	0	0	2	5(100)	10
Kerman (Device: n=9; air: n=3)	*Trichothecium*	1						1(16.7)							0	1(14.2)
	*Aspergillus spp.*				1		1	2(33.3)							0	2(28.5)
	*Rhizopus*	2						2(33.3)	1						1(100)	3(42.8)
	*Penicillium*			1				1(16.7)							0	1(14.2)
	Total	3	0	1	1	0	1	6(100)	1	0	0	0	0	0	1(100)	7
Birjand (Device: n=9; air: n=0)	*Alternaria*	1						1(33.3)							0	1(33.3)
	*Aspergillus spp.*			1	1			2(66.6)							0	2(66.6)
	Total	1	0	1	1	0	0	3(100)							0	3
Arak (Device: n=9; air: n=3)	*Aspergillus spp.*			1		3		4(100)					4		4(100)	8(100)
	Total	0	0	1	0	3	0	4(100)	0	0	0	0	4	0	4(100)	8
Bandar Abbas (Device: n=9; air: n=3)	*Rhizopus*	2						2(50)							0	2(25)
	*Aspergillus spp.*			2				2(50)	4						4(100)	6(75)
	Total	2	0	2	0	0	0	4(100)	4	0	0	0	0	0	4(100)	8
Mashhad (Device: n=9; air: n=0)	*Rhizopus*		1					1(33.3)							0	1(33.3)
	*Aspergillus spp.*	1					1	2(66.6)							0	2(66.6)
	Total	1	1	0	0	0	1	3(100)							0	3

Out of 210 isolated colonies, the rate of isolated colonies based on the sampled wards were as follows: ICU (38.5%; 29.5% device and 9% air), operating room (21.9%; 19.5% device and 2.4% air), oncology (15.2%; 10.9% device and 4.3% air), general ward (13.4%; 10.5% device and 2.9% air), neonatal (5.7%; 4.3% device and 1.4% air), and NICU (5.3%; 2.4% device and 2.9% air). 

**Figure 1:**
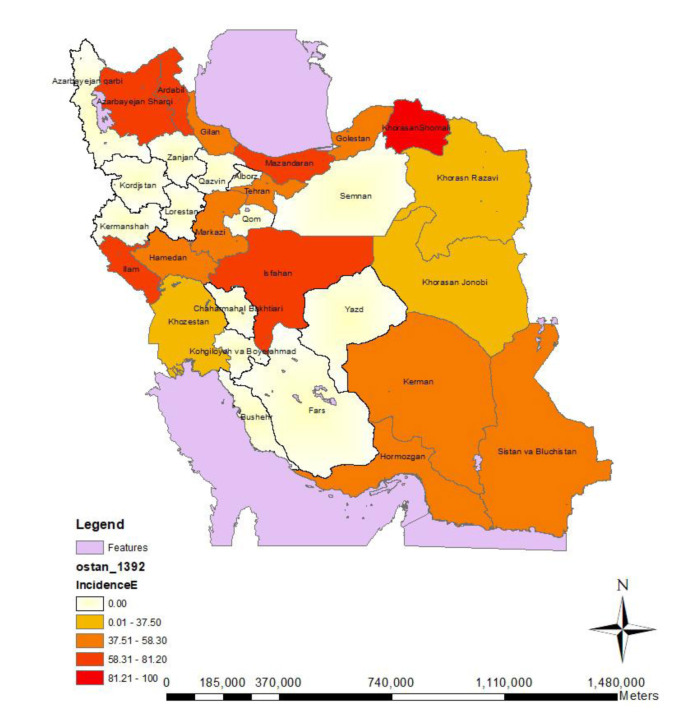
Fungal contamination rates in the hospitals located in different provinces of Iran

Figure 1 shows the fungal contamination rate of each hospital from different parts of Iran. Out of 256 collected samples from equipment and air, 163 (63.7%) were positive for fungal growth. The rate of fungal colonization in instrument and air samples was 128/213 (60.1%) and 35/43 (81.2%), respectively. Bojnurd has the highest contamination rate (100%), followed by Mazandaran and Isfahan provinces, with rates of 81.2% and 78.9%, respectively. The lowest contamination rate was related to Dezful, Birjand (33.3% each), and Mashhad (22.2%). 

**Figure 2:**
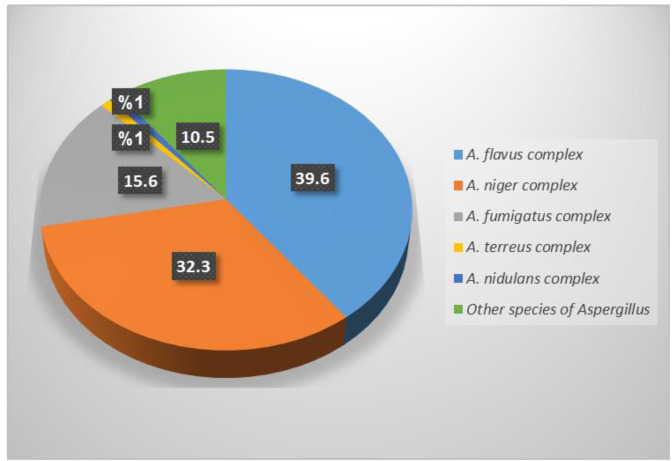
Distribution of different species of *Aspergillus* in the studied hospitals

Among 96 isolates of *Aspergillus*, *A. flavus* complex (38/96, 39.6%), A. niger complex (31/96, 32.3%), and *A. fumigatus* complex (15/96, 15.6%) were the commonest species (Figure 2). 

**Figure 3:**
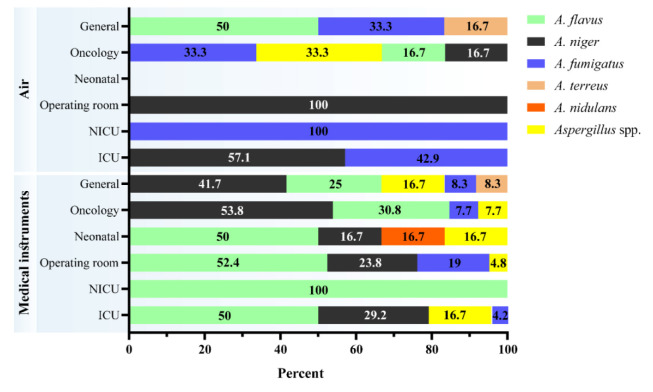
Distribution of different species of *Aspergillus* in the studied hospitals based on the collected samples from different hospital wards

Among different isolated species of the *Aspergillus* from the surface samples, *A. flavus* complex had the highest rate of isolation in ICU (12/24, 50%), NICU (1/1, 100%), operating room (11/21, 52.4%), and neonatal wards (3/6, 50%), whereas *A. niger* complex was more prevalent in oncology (7/13, 53.8%) and general wards (5/12, 41.7%). In the air samples, *A. niger* complex had the highest frequency in ICU (4/7, 57.1%) and operating room (1/1, 100%); moreover, the highest frequency of *A. fumigatus* complex was observed in NICU (1/1, 100%) and ICU (3/7, 42.9%). However, *A. flavus* complex showed the highest isolation rate in the general ward (3/6, 50%) (Figure 3).

## Discussion

Aspiration of airborne fungal conidia has been significantly considered to cause infection, and hence, increasing exposure to fungal contamination sources including airborne or deposited fungal propagules on different medical equipment in the vicinity of the susceptible hospitalized hosts is a serious issue [ 15 ]. According to this reality, this multi-center study evaluated the indoor environment of different educational hospitals located in different parts of Iran in order to detect fungal contamination sources. 

In this present study, among different fungal genera isolated from surfaces and air samples, *Aspergillus* was the most abundant fungi. However, in contrast to instrument samples, *Cladosporium* had the second rank after *Aspergillus* in air samples, whereas in instrument samples, *Rhizopus* and *Mucor* were the most prevalent genera after *Aspergillus*. *Cladosporium* and some species of *Aspergillus* in comparison to *Rhizopus* and *Mucor* have smaller conidia. Interestingly, *Aspergillus fumigatus*, which has smaller conidia, compared to *A. flavus*, was more common in air samples. Unlike, *A. flavus* was a more abundant species of *Aspergillus* in instrument samples. The smaller size of conidia allows the respective fungi to remain in the air for a long time before setting on the surface [ 16 ]. In our previous experience, *Cladosporium*, *Aspergillus*, and *Peni-cillium* were the most common isolates in hospital air samples and with less frequency for *Aspergillus* in equipment [ 17 ]. In line with our findings, in a recently conducted study from Iran, *Aspergillus*, *Penicillium*, and *Cladosporium* were the most abundant mold in air and instrument samples [ 18 ]. In addition, several recent reports in different hospitals from different parts of Iran have shown that Aspergillus species are among the most predominant isolates in air samples [ 19 - 21 ].

However, in some reports from Iran [ 22 ] and Turkey [ 23 ], the genus *Aspergillus* was not identified as the most prevalent fungi in the indoor air of hospital wards. It is worth to be noted that in our study, the *Aspergillus* genus was also the most predominant mold in most hospitals, especially in instrument-collected samples, while in Tehran and Tabriz, *Mucor* and *Rhizopus* genera were the prevalent isolates, respectively. 

In this present study, based on the sampled hospital wards, ICU and operating rooms had the highest number of isolated fungal colonies from instrument and surface collected samples. *Aspergillus* genus was also reported as the most prevalent in all wards, except for NICU. These findings become more important when it is noted that much evidence has shown the importance of the presence of *Aspergillus* in the special wards with immunocompromised, high risk, and ill patients, including ICU, to put the patient at risk for life-threatening infections [ 2 , 3 , 13 ]. On the other hand, long stays in ICU can provide a more suitable opportunity to expose the patients to indoor fungi and increase the risk of fungal infections.

 In this present study, among different species of *Aspergillus*, *A. flavus* complex was the most predominant isolate, followed by *A. niger* complex and *A. fumigatus* complex. Similar to our findings, several studies from different parts of Iran reported *A. flavus* as the prevalent species of *Aspergillus* in the air and equipment of hospital samples [ 24 , 25 ]. Although in some reports, *A. flavus* was ranked second after *A. niger* [ 19 , 21 , 26 ]. According to our previous experiences, *A. flavus* has interestingly been the most prevalent species of *Aspergillus* found in different clinical and environmental samples in Iran [ 27 - 29 ]. It is suggested that the genetic characteristics of this species for geographical adaptation to hot and dry climates are the possible reason for the higher incidence of *A. flavus* in the Middle East, including Iran [ 30 , 31 ]. It is also noted that the local predominance of *A. flavus* indicates a consistent relationship between *Aspergillus* distribution and temperature [ 32 ].

In this present study, the rate of fungal contamination in the air samples was higher than that of surface and equipment which shows a comparable result with other studies [ 19 , 21 , 24 - 26 ]. Surface and equipment in hospitals have mainly been considered for the study of bacterial contamination; however, the findings in our study are in line with those of some previous reports [ 7 , 33 ] highlighting the importance of deposited fungal spores on surfaces and instruments as one of the probable sources of fungal contamination in the indoor environment of hospitals.

## Conclusion

According to our findings, in addition to air, equipment and instrument should be considered among the significant sources of fungal contamination in the indoor environment of hospitals. Based on the high rate of fungal contamination in the collected samples, the importance of monitoring and control measures of the indoor environment of the hospital are emphasized.

## Acknowledgments

The research reported in this publication was supported by Elite Researcher Grant Committee under award number (983110) from the National Institute for Medical Research Development (NIMAD), Tehran, Iran.

## Authors’ contribution

M.T.H. was involved in the concept and design of the study. M.G. and M.T.H. wrote the main manuscript text. J.Y.C. did statistical analyses. N.K., M.K., F.K., Y.N., B.R., M.H.M., B.S., S.A.J., M.D., A.S., S.A.S., N.A., M.N., A.G., K.A., M.A., M.N., M.R.Z., S.H., and H.H., performed the sampling and primary fungal isolation. M.A., I.H., M.G., and M.T.H. did fungal identification, review, and editing of the manuscript. All authors reviewed the manuscript.

## Conflicts of interest

The author(s) declared no potential conflicts of interest with respect to the research, authorship, and/or publication of this article.

## Financial disclosure

No financial interests related to the material of this manuscript have been declared.
